# Evolution or Revolution? AI in Coronary CT Evaluation

**DOI:** 10.1016/j.jacadv.2024.100860

**Published:** 2024-03-06

**Authors:** Kelley R.H. Branch

**Affiliations:** Division of Cardiology, University of Washington Heart Institute, Seattle, Washington, USA

**Keywords:** artificial intelligence, atherosclerosis, coronary artery disease, coronary plaque, high risk plaque, machine learning

**Figure:**
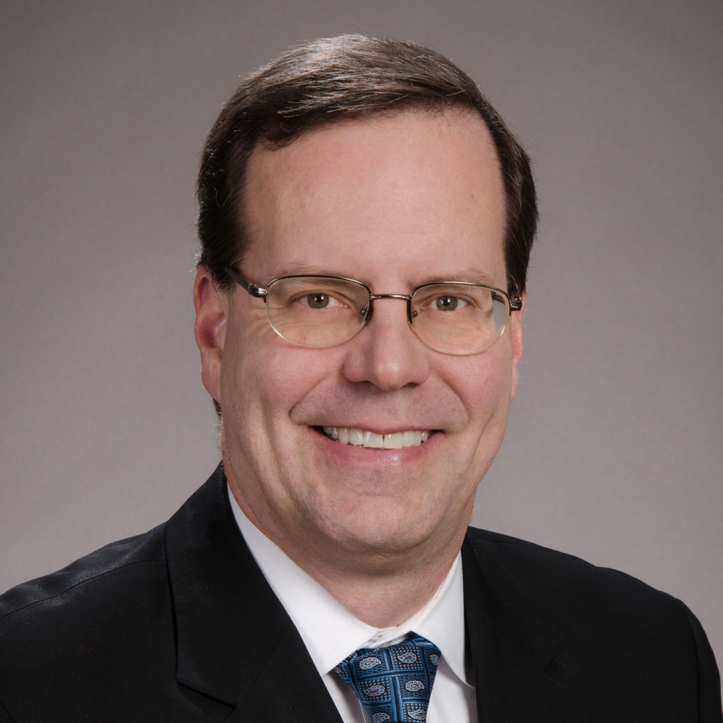

Coronary computed tomography angiography (CCTA) has made huge strides over the past 20+ years, from a niche, somewhat cumbersome imaging modality to a rapid, highly accurate, Level 1A guideline recommendation for chest pain evaluation. However, with rapid growth come precipitous challenges. At present, there are limited expert readers in CCTA, and that number will only get worse with the expected explosion of CCTA ordering over the coming years.^1^ With current training requirements and the need for hundreds of mentored CCTA scans to achieve competency, there is no way to easily fill the void.^2^ This is further exacerbated by the increasingly more complex disease evaluated with CCTA, including plaque burden and high-risk plaque features, which has manifested into an urgent need for robust yet streamlined computed tomography (CT) coronary evaluation. A potential solution is the application of artificial intelligence (AI) and machine learning to CCTA for both coronary stenosis and atherosclerotic plaque analysis. A number of recent studies showed that application of AI for CCTA evaluation had good diagnostic accuracy and correlation with expert readers or invasive coronary angiography (Table 1).

**Table 1:** Per-Vessel Level Data for AI Analysis of CCTA (All Stenosis)

First Author	Year	N/No. of Vessels	Comparator	AI Maker/Type	Diagnostic Accuracy	Sens	Spec	Correlation or Agreement^a^
Choi^4^	2021	232/924	Level 3 readers	CLEERLY	--	77 (--)	98 (--)	R = 0.92
Khasanova^5^	2022	50/200	Level 3 readers	HeartFlow	--	--	--	L3 k = 0.46 AI k = 0.71
Yi^6^	2022	71/213	ICA	CoronaryDoc	80 (74-85)	74 (65-82)	86 (77-91)	k = 0.39
Liu^7^	2021	165/680	Level 1-3 readers, ICA	CoronaryDoc	89 (87-91)	81 (76-86)	94 (81-96)	AUC = 90
Griffin^8^	2023	303/848	ICA	CLEERLY	--	--	--	R = 0.73
Ihdayhid^3^	2024	769/3,012	Level 3 reader	CNN	90 (88-91)	91 (89-92)	89 (87-90)	AUC = 90

AI = artificial intelligence; AUC = receiver operator curve area under the curve; CNN = convolutional neural network; k = kappa agreement; R = Pearson’s coefficient; Sens = sensitivity; Spec = specificity.

a Interobserver agreement.

In this issue of *JACC: Advances*, Ihdayhid et al^3^ present a fully automated, unsupervised AI application to evaluate coronary artery stenosis as well as high-risk plaque characteristics with CCTA. This study from 4 centers developed a deep learning AI algorithm with training and validation cohorts, followed by 769 patients (with 3,012 coronary arteries) for the testing cohort. Obstructive coronary artery disease (CAD) (>50% stenosis and CAD Reporting and Data System 3-5) was present in 35% of patients. Overall agreement with per vessel and per patient analyses was good at 71% and fair at 55.5%, respectively, with better agreement for obstructive CAD of 93.6% and 87.1%, respectively. This supports data from the other studies, where the highest discrepancies were in diagnosis of nonobstructive (<50% stenosis) CAD. It is important to point out that the current study did not require any human input, unlike the prior “semi-automated” AI studies. These data also compare favorably to prior studies, including the similarly designed CLARIFY study^4^ (Table 1). In the smaller number of 45 patients with high-risk plaque features identified by expert level 3 readers, diagnostic accuracy ranged from 84% to 90% for the 20% of vessels with high risk plaque. The total time required for AI coronary analysis was approximately 2 minutes.

The question is whether we are witnessing a revolution or the more measured, fussy evolution of AI technology for CCTA. The data strongly suggest the latter. The current study is similar to the other studies in that AI is no panacea—the AI CCTA diagnostic accuracy measures remain <100% and are quite variable whether one considers patient, coronary vessel or segments, or when considering all stenosis levels or only obstructive CAD (>50% or >70%). While the numbers in Ihdayhid et al^3^ are comparatively large for an imaging study, they are somewhat limited for the many thousands of data points often needed for optimal AI training. This is especially true of the high-risk plaque, where the small number of patients and coronary arteries studied (N = 45 and N = 325, respectively) were likely underpowered for robust conclusions. Further, the current study also only used one level 3 reader for each study, which could affect the ground truth and thus the accuracy of data, for better or for worse. Fortunately, the data appear within range of prior studies suggesting utility of the current algorithm, but with some caution.

When considering Ihdayhid et al^3^ and the other prior studies, some truths and future directions become clear. First, current iterations of AI CCTA for stenosis appear to be similar but not superior to expert readers such that the net benefit is primarily the rapidity of quantitative information. Given the expected limited expert readers in the coming years and the need for efficiency, this is a desirable benefit. Second, AI is still subject to the limitations of CT for nonevaluable scans and segments (3% of patients and an unknown number of vessels/segments in the current study) and it is likely CT technologic evolution and not AI that will affect this favorably. Third, CCTA AI data are generally compared to “bronze” standards of invasive coronary angiography and human Level 3 CCTA readers with their well-known, well-described intraobserver and interobserver variabilities. This is especially true with total plaque and high-risk plaque features. This makes a “ground truth” somewhat elusive although pathologic studies and planned studies with multimodality imaging, including intracoronary imaging, with the INVICTUS imaging registry study (NCT04066062), will provide reassurance. Fourth, despite the Standards for Reporting of Diagnostic Accuracy Studies requirements, there is a lack of uniform reporting of diagnostic accuracy measures for patient and vessel level and for all stenoses and as categorical stenoses in the current and prior reports (Table 1). This makes comparison between the different types of AI nearly impossible. Guidelines for reporting AI CCTA data are clearly needed and their development and subsequent adoption are an anticipated and much needed part of the evolution. Finally, implementation of AI may be time-saving, but needs to be balanced against the potential costs of technology, especially if an additional cost burden per scan to the patient. The lure of efficiency is strong but must be balanced into a cost-benefit analysis.

Over a relatively short period of time, AI/machine learning application to CCTA evaluation has shown great promise to make coronary imaging more efficient for a range of reader expertise and for the type and degree of CAD. Without the application of streamlined CCTA analysis with AI or other technologies, it is unlikely that imagers will be able to meet the demand for CCTA in the coming years. Studies like those provided by Ihdayhid et al^3^ are critical to our trust and adoption of the many AI applications for coronary analysis, but there is clearly additional work to do. Regardless, this is yet one more step in the evolution toward the seeming eventual revolution of CCTA evaluation with AI.

## Funding support and author disclosures

Dr Branch has industry relationships with Bayer, Amgen, Novartis, Hanmi, Kestra, and Janssen.
